# Learning a Continuous Progression Trajectory of Alzheimer’s disease for Personalized Tracking

**DOI:** 10.64898/2026.02.03.703568

**Published:** 2026-02-06

**Authors:** Mingzhao Tong, Fahad Mehfooz, Shu Zhang, Yipei Wang, Shiaofen Fang, Andrew J. Saykin, Xiaoqian Wang, Jingwen Yan

**Affiliations:** a Department of Computer Science, Indiana University Indianapolis, Indianapolis, IN, 46202, U.S.; b Department of Biomedical Engineering and Informatics, Indiana University Indianapolis, Indianapolis, IN, 46202, U.S.; c Department of Computer Science, University of California Los Angeles 404 Westwood Plaza Engineering IV, Los Angeles, CA, 90095, U.S.; d Elmore Family School of Electrical and Computer Engineering, Purdue University, West Lafayette, IN, 47906, U.S.; e Department of Radiology and Imaging Sciences, Indiana University School of Medicine, Indianapolis, IN, 46202, U.S.

**Keywords:** Alzheimer’s disease, Progression modeling, Early detection

## Abstract

**BACKGROUND:**

Understanding of Alzheimer progression is critical for timely diagnosis and treatment evaluation, but traditional discrete diagnostic groups often lack sensitivity to subtle early-stage changes.

**METHODS:**

We developed SLOPE, an unsupervised dimensionality reduction method that models the amyloid progression in AD on a continuous scale while preserving the temporal order of longitudinal follow-up visits. Applied to longitudinal amyloid PET data, SLOPE generated a two-dimensional trajectory capturing global amyloid accumulation across the AD continuum.

**RESULTS:**

SLOPE-derived staging scores better preserved temporal progression across diagnostic groups and longitudinal follow-up visits and can be generalized to held-out subjects. The learned trajectory revealed biologically consistent amyloid spreading patterns and greater sensitivity to early progression than global amyloid SUVR.

**DISCUSSION:**

SLOPE provides a continuous staging of amyloid pathology that complements global amyloid measures by capturing early localized progression. These properties highlight its potential in disease modeling and monitoring, particularly in early and preclinical stages of AD.

## 1. Background

Alzheimer’s disease (AD) is a progressive irreversible neurodegenerative disorder characterized by the accumulation of amyloid-β plaques. Clinically, AD unfolds over decades, typically beginning with subtle cognitive changes that gradually worsen to noticeable impairments in memory and daily functioning [1]. Despite a few FDA-approved drugs, they offer only symptomatic relief and no definitive cure exists currently [2]. In this context, accurately modeling disease progression and detecting subtle early-stage changes are critical for timely diagnosis, thereafter improving assessment of treatment effectiveness. One of the main challenges in studying AD progression lies in the nature of observational cohort data. Due to the extended timeline of disease development, most longitudinal studies capture only a limited snapshot of each individual’s disease course. As a result, the full temporal dynamics of AD remain difficult to reconstruct. This restricted temporal resolution hinders efforts to develop accurate models of disease progression and to identify the earliest pathological changes that could serve as targets for intervention.

Traditional analyses often focus narrowly on categorizing individuals into coarse diagnostic groups, such as cognitively normal (CN), mild cognitive impairment (MCI), and Alzheimer’s disease (AD) patients [3]. While this stratification offers a convenient framework for broadly describing the course of AD, these coarse groupings are not enough to capture the continuous course of AD. Especially in the early stage, individuals were often labeled as cognitively normal or amyloid-negative without further differentiation due to the absence of noticeable symptoms [4]. This oversimplification masks subtle yet clinically meaningful changes and reduces the sensitivity of models to detect early transitional states. It sets a critical barrier to our understanding of early pathological changes in AD, which are critical for timely diagnosis and intervention.

In recent years, pseudotime-based approaches have emerged as powerful tools to model the progression trajectories of diseases. These methods aim to infer an underlying temporal order of samples based on high-dimensional biomarker data, capturing latent disease progression patterns in the absence of explicit time stamps. Pseudotime analysis was originally developed to model the dynamic process of cell differentiation from single-cell data and has proven to be successful in capturing both the transition of cell states and the associated molecular changes [5–7]. Recently, researchers also found its great potential in modeling the progression of disease using gene expression and brain imaging data, in which pseudotime models attempt to reconstruct the underlying sequence of pathological changes across individuals at different disease stages in a continuous manner [8–10].

Despite their promise, pseudotime methods have notable limitations. They are primarily non-parametric and often used for exploratory discovery rather than prediction tasks, with very limited generalizability to new or unseen patients. Moreover, these models are typically applied to cross-sectional data and lack mechanisms to incorporate known temporal context from longitudinal follow-up visits, which is critical for tracking disease progression in real-world clinical settings. Finally, the absence of standardized benchmarks or widely accepted evaluation criteria for validating estimated disease trajectories makes it challenging to assess their reliability and reproducibility, thereby limiting broader application in clinical research.

To address these challenges, we present a novel Self-supervised Longitudinal Progression Embedding (SLOPE) method for modeling Alzheimer’s disease progression trajectories. Unlike traditional classification models or purely unsupervised pseudotime approaches, SLOPE integrates both high-dimensional biomarker data and temporal context from longitudinal follow-up visits. This parametric framework produces a visually intuitive 2D progression trajectory, enabling individualized progression monitoring for both training and unseen patients. In addition, we proposed two new metrics to evaluate the robustness of the estimated progression trajectory. Given that amyloid accumulation is considered one of the earliest pathological features of AD [11], we applied the SLOPE method to longitudinal amyloid PET imaging data from the Alzheimer’s Disease Neuroimaging Initiative (ADNI), a rich and temporally diverse dataset spanning the full spectrum of AD progression. Compared to state-of-the-art methods, SLOPE more accurately recovered the temporal ordering of follow-up visits. In addition, SLOPE-derived amyloid staging score showed greater sensitivity to early regional amyloid accumulation than global amyloid SUVR, highlighting its great potential as a complementary framework in modeling and tracking Alzheimer’s disease progression.

## 2. Methods

### 2.1 Dataset

Data used in the preparation of this article was obtained from the Alzheimer’s Disease Neuroimaging Initiative (ADNI) database (adni.loni.usc.edu). The ADNI was launched in 2003, aimed to test whether multi-modal neuroimaging, other biological markers, and clinical and neuropsychological assessment can be combined to measure the progression of mild cognitive impairment (MCI) and early Alzheimer’s disease (AD). The current goals include validating biomarkers for clinical trials, improving the generalizability of ADNI data by increasing diversity in the participant cohort, and providing data concerning the diagnosis and progression of Alzheimer’s disease to the scientific community.

Longitudinal Amyloid PET data was downloaded from the ADNI website, which was processed by the UC Berkeley group and had already undergone pre-processing steps, providing direct regional amyloid standardized uptake value ratios (SUVRs) for each brain region. A detailed description of the pre-processing pipeline, including image alignment, intensity normalization, and SUVR estimation, is available in the corresponding documentation provided by ADNI (https://adni.loni.usc.edu/data-samples/adni-data/neuroimaging/pet/).

This dataset includes florbetapir amyloid PET data from 961 individuals across 2,023 visits; data from other amyloid PET tracers were excluded to ensure consistent SUVR scaling and regional comparability. Each visit (i.e., data point) is classified into one of four diagnostic groups: cognitively normal (CN), early mild cognitive impairment (EMCI), late mild cognitive impairment (LMCI), or Alzheimer’s disease (AD). Our analysis of amyloid PET focused on the standardized uptake value ratio (SUVR) across 68 cortical regions of interest (ROIs), which indicates the amyloid-β uptake levels. These regional SUVR values were normalized using COMPOSITE REF SUVR as the reference region, a summary measure provided by ADNI. This normalization step helps control for nonspecific binding and individual variability, ensuring that measurements accurately reflect true amyloid deposition. Subcortical regions were excluded from the amyloid analysis, as their amyloid burden is less specific and not closely associated with Alzheimer’s disease. To account for potential biases related to age, gender and years of education, all imaging measures were pre-adjusted using weights derived from cognitively normal individuals at baseline. Detailed demographic information is provided in Table 1. All imaging features were standardized to have a mean of zero and a standard deviation of one for subsequent analysis. This center-scaling of amyloid SUVR values was applied solely to improve the training stability of proposed SLOPE model and does not alter the biological interpretation of amyloid positivity, as the model learns relative regional amyloid patterns rather than relying on absolute SUVR thresholds.

**Figure 1:**
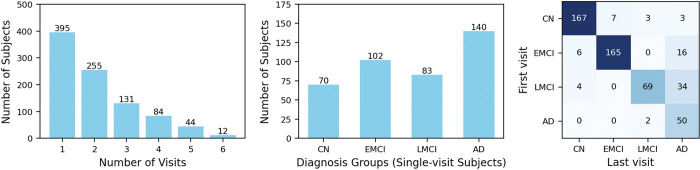
Distribution of ADNI participants with amyloid-PET data. Left: number of follow-up visits among all participants. Middle: Diagnosis distribution of participants with only baseline visit. Right: diagnosis distribution of participants with multiple follow-up visits. Heatmap represents the diagnosis change from baseline to last visit.

In total, there are 395 individuals with only baseline visits and 526 individuals with multiple follow-up visits (Fig. 1.) Among those with only baseline data, there are 70 CNs, 102 EMCIs, 83 LMCIs, and 140 AD patients. For individuals with multiple follow-up visits, this data set provides broad temporal coverage, capturing transitions across different disease stages, such as CN to EMCI and EMCI/LMCI to AD. All individuals, regardless of whether they had follow-up visits, were included in the trajectory learning and evaluation.

### 2.2 Self-supervised Longitudinal Progression Embedding (SLOPE)

**Figure 2:**
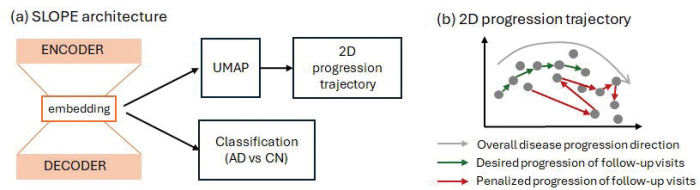
Overview of the proposed approach (a). SLOPE model will generate a low-dimensional embedding that preserves the temporal order of each individual’s follow-up visits. This embedding will be further reduced into 2D via UMAP, which is expected to preserve the temporal order of follow-up visits learned from SLOPE and form a 2D disease progression trajectory, reflecting the continuous process of amyloid accumulation in the brain (b).

We employed a two-step process to derive a compact and smooth two-dimensional trajectory embedding, where all follow-up visits were temporally ordered along a principal curve. First, we developed the Self-supervised Longitudinal Progression Embedding (SLOPE) model, which reduces the dimension of original regional amyloid SUVR values while explicitly preserving the temporal sequence of each individual’s follow-up visits. Second, the resulting embeddings from SLOPE were further mapped into a two-dimensional space, enabling visual tracking of disease progression. Fig. 2a provides an overview of the proposed SLOPE model, while Fig. 2b illustrates the target two-dimensional disease progression trajectory. The goal is for each subject’s follow-up visits to generally align with the overall progression direction, with minimal reversals or abrupt changes along the trajectory.

SLOPE is designed as an autoencoder-based neural network to learn a low-dimensional embedding of original regional amyloid-β levels (Fig. 2a). This embedding is expected to form a compact, continuous trajectory curve that captures the progression of amyloid profiles across the course of AD development (Fig. 2b). Unlike traditional supervised approaches, SLOPE is trained in a fully self-supervised manner, without relying on diagnostic labels. The model architecture consists of two main components:

- **Encoder** $g_{\phi }$: maps the amyloid SUVR values of 68 brain regions for subject i at visit k, denoted as $x_{i,k}$, into a low-dimensional embedding $z_{i,k}$.
- **Decoder** $g_{\phi }^{-1}$: reconstructs the original amyloid SUVR vector from the embedding.

The autoencoder is trained by minimizing a reconstruction loss that measures the discrepancy between the observed amyloid profile and the decoder’s output:

$$\;z_{i,k}=g_{\phi }\left(x_{i,k}\right)\;L_{rec}=\sum_{i,k}\;{\left\|x_{i,k}-g_{\phi }^{-1}\left(z_{i,k}\right)\right\|}^{2}$$

To account for longitudinal follow-up data, we introduce an additional penalty term, namely the direction loss, which enforces a biologically plausible progression trajectory. Specifically, this loss constrains the latent trajectory of each individual so that their amyloid accumulation either remains stable or increases across visits, reflecting the assumption that amyloid accumulation in Alzheimer’s disease does not spontaneously reverse in the absence of intervention. Formally, we define the vector ${\vec{v}}_{i,k}$ to represent the progression direction between consecutive visits. Here, $z_{i,k}$ and $z_{i,k+1}$ are the low-dimensional embeddings of the subject i at visits k and k+1, respectively. Intuitively, ${\vec{v}}_{i,k}$ represents the direction of change in amyloid burden over time in the embedding space.


$${\vec{v}}_{i,k}=z_{i,k+1}-z_{i,k}$$

Direction loss $L_{\mathrm{dir}}$ penalizes the inconsistencies in progression vectors within each subject. Specifically, it encourages consecutive progression directions (${\vec{v}}_{i,k}$ and ${\vec{v}}_{i,k+1}$) to remain aligned by maximizing their cosine similarity. Sharp directional changes across a subject’s follow-up visits reduce cosine similarity, leading to a higher penalty. As a result, such abrupt changes as demonstrated in Fig. 2b are discouraged and penalized during the training process.


$$L_{dir}=-\sum_{k<K_{i}}\;\left(1-\cos\left(v_{i,k},v_{i,k+1}\right)\right)$$

$$\cos({\vec{v}}_{i,k},{\vec{v}}_{i,k+1})=\frac{{\vec{v}}_{i,k}\cdot {\vec{v}}_{i,k+1}}{\left\|{\vec{v}}_{i,k}\right\|\left\|{\vec{v}}_{i,k+1}\right\|}$$

The training process of SLOPE aims to control the reconstruction loss and direction loss simultaneously, by minimizing the weighted total loss $L_{total}=\lambda_{1}L_{rec}+\lambda_{2}L_{dir}$. Here, $\lambda_{1}$ *and* $\lambda_{2}$ are two hyperparameters that control the relative contribution of two losses. The reconstruction loss preserves similarity among individuals with comparable amyloid profiles in the embedding space, while the direction loss enforces smooth progression along the sequence of follow-up visits, thereby preserving their temporal ordering. The direction loss was applied only to subjects with two or more longitudinal visits, whereas subjects with a single visit contributed exclusively to the reconstruction loss and did not influence temporal ordering constraints. Together, minimizing these two losses encourages the embedding to form a smooth and compact trajectory that captures both cross-sectional similarity across subjects and the longitudinal structure of disease progression.

#### 2.2.1 Training and Test Data Split

The entire amyloid-PET dataset was split at the subject level to prevent any potential data leakage between the training and test sets. To preserve the distribution of diagnostic groups across both subsets, we employed stratified random sampling with a fixed random seed. This ensured that 80% of unique subjects were assigned to the training set and the remaining 20% to the test set. To ensure a fair comparison, the same data partition was applied across all competing methods.

#### 2.2.2 Hyperparameter Optimization

Model hyperparameters were optimized using a hybrid tuning strategy. Grid search was first applied to explore discrete architectural parameters, such as the number of layers, hidden dimensions, and activation functions. This was followed by random search to fine-tune continuous parameters like the learning rate and regularization strengths (i.e., $\lambda_{1}$ and $\lambda_{2}$). For supervised baseline models, 5-fold cross-validation was performed for hyperparameter tuning within the training set to ensure stable performance and mitigate overfitting.

In contrast, SLOPE and the traditional autoencoder are self-supervised models that do not use diagnostic labels during training. These models serve primarily as unsupervised dimensionality reduction techniques, and therefore, the typical goals of cross-validation, such as estimation of prediction performance or supervised parameter tuning, do not directly apply. Like PCA, both SLOPE and autoencoder learn a transformation of the input data exclusively from the training set, and this transformation is subsequently applied to the test set, which remains entirely unseen during model training and hyperparameter tuning. The best SLOPE model was trained for 1,550 epochs using a five-layer architecture with dimensions [68, 286, 20, 286, 68] and ReLU activation. Optimization was performed with the Adam optimizer and a learning rate of 2.44 × 10^−5^, minimizing mean squared error (MSE) as the loss function. The model balanced its objectives using a reconstruction weight ($\lambda_{1}$) of 1.0. In summary, the best SLOPE model generated a 20-dimensional latent embedding for each visit. This embedding was subsequently projected to two dimensions using UMAP for visualization. UMAP was used solely for visualization of the learned latent embeddings and was not part of SLOPE model training. Using the umap-learn package, UMAP was trained in an unsupervised manner on the training-set embeddings and the learned transformation was applied to the test subjects using default parameters without hyperparameter optimization.

### 2.3 Pseudotime calculation

The low-dimensional embeddings learned from SLOPE were further transformed using a parametric UMAP model, resulting in a two-dimensional representation of the progression trajectory. Within this 2D space, we fitted a principal curve that traces the overall direction of progression trajectory. Amyloid data from each visit of training subjects was then projected onto this curve, and its relative position was computed using the SlingShot package [4]. These positions were subsequently normalized to the range [0,1], yielding a continuous measure (namely pseudotime) that reflects the global amyloid burden across the brain. Once the SLOPE and UMAP models were trained, the same procedure was applied to previously unseen test subjects. Specifically, the regional amyloid SUVR values from each visit were passed through the pre-trained SLOPE and UMAP models and then projected onto the 2D progression trajectory. This projection enabled us to assign a pseudotime value to each visit, representing its position along the full spectrum of amyloid progression.

### 2.4 Model evaluation

We evaluated the SLOPE model from three perspectives: 1) whether the derived pseudotime reconstructs the progressive amyloid severity of diagnosis groups? 2) whether it preserves the temporal order of follow-up visits? and 3) whether its embeddings improve downstream classification compared to raw amyloid SUVR values? SLOPE is benchmarked against a traditional autoencoder, which similarly yields low-dimensional embeddings and pseudotime, as well as supervised models including logistic regression, elastic net regression, and multilayer perceptron (MLP). These supervised models were trained to discriminate AD from CN, yielding a continuous logit score for each visit, which was normalized into [0,1]. We compare these logits with pseudotime from SLOPE and the autoencoder in terms of their ability to preserve the temporal sequence across diagnosis groups and follow-up visits. Progression across diagnosis groups was assessed by comparing the distributions of pseudotime (for SLOPE and autoencoder) or logits (for supervised models) across diagnosis groups, with statistical significance computed from t-test. Temporal consistency across follow-up visits was evaluated using two metrics: the violation ratio, which measures the frequency of reversals, and the violation gap, which quantifies the severity of these reversals.

The violation ratio r measures the proportion of consecutive (neighbor) pairs of follow-up visits where the expected progression is violated. Under the assumption of irreversible amyloid accumulation, we expect the amyloid severity score (i.e., pseudotime) at the current visit to be the same as or lower than that at the next visit. If the estimated pseudotime values of these two visits show the opposite pattern (i.e., a reduction in amyloid burden during follow-up visits), this is considered a direct violation. The violation ratio thus reflects how consistently the model preserves forward progression across consecutive visits. A lower violation ratio indicates better preservation of temporal order in follow-up visits, and it is formulated as:

#(1) $$r=\frac{1}{N}\sum_{i=1}^{N}\;\sum_{k=1}^{K_{i}-1}\;1\left[p_{i,k+1}<p_{i,k}-\tau \right]$$

Here, N is the total number of subjects, $K_{i}$ is the number of visits for subject $i,p_{i,k}$ is the pseudotime value for subject i at visit k, and 1[⋅] is the indicator function that equals 1 if the condition is true and 0 otherwise. The violation tolerance parameter τ allows small reversals of pseudotime between follow-up visits to be ignored when assessing temporal ordering.

In addition to the violation ratio, we introduce the violation gap g as a complementary metric. It is defined as the average pseudotime difference among consecutive visit pairs with direction violation. The violation gap therefore reflects the severity of reversals, rather than just their frequency. Larger violation gaps indicate more pronounced inconsistencies in the direction of disease progression across follow-up visits, reflecting the poor reliability of the model’s disease progression estimates.

#(2) $$g=\frac{\sum_{i=1}^{N}\;\sum_{k=1}^{K_{i}-1}\;1\left[p_{i,k+1}<p_{i,k}-\tau \right]\cdot \left|p_{i,k+1}-p_{i,k}\right|}{\sum_{i=1}^{N}\;\sum_{k=1}^{K_{i}-1}\;1\left[p_{i,k+1}<p_{i,k}-\tau \right]}$$

In addition, we compared the classification performance of the low-dimensional embeddings derived from SLOPE and the autoencoder with that of the original regional SUVR features. For each feature set, three supervised classifiers were trained using 5-fold cross-validation within the training set, and their performance was subsequently evaluated on the held-out test set. Model performance was assessed using three metrics: balanced accuracy across diagnostic groups, area under the ROC curve (AUC), and F1 score. Both balanced accuracy and F1 score are particularly useful for evaluating classification performance in the presence of class imbalance, as they account for unequal group sizes and the trade-off between precision and recall.

### 2.5 Comparison of SLOPE Pseudotime with Composite Amyloid SUVR

To examine whether a temporally preserved amyloid staging axis captures progression beyond global amyloid burden, we performed three analyses comparing SLOPE pseudotime with composite amyloid SUVR. First, we assessed differences between CN and EMCI participants using both measures, performing analyses separately in the training and held-out test sets to evaluate generalization. Second, we examined the relationship between composite amyloid SUVR and SLOPE pseudotime using two-segment piecewise linear regression, allowing for nonlinear associations along the staging axis. Third, to assess whether early changes in pseudotime reflect biologically meaningful signals, we evaluated associations between early pseudotime values and regional amyloid SUVRs across 68 cortical regions.

## 3. Results

### 3.1 Progression trajectory

**Figure 3:**
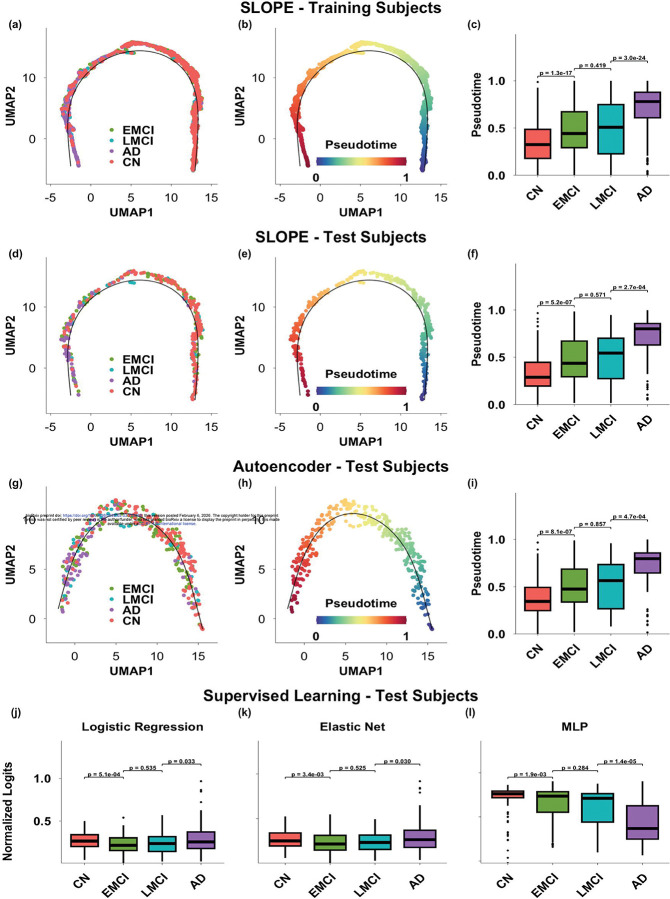
Performance comparison of SLOPE and baseline methods in preserving progressive pattern across diagnosis groups and follow-up visits. a–b: SLOPE-derived progression trajectories for training subjects, colored by diagnosis group (a) and pseudotime (b). c: Pseudotime distribution across all visits of training subjects, stratified by diagnosis group. d–f: Test subjects projected onto the SLOPE-derived progression trajectory and their pseudotime distribution. g–i: progression trajectory and pseudotime distribution derived from traditional autoencoder. j-l: Distribution of normalized logits across diagnosis groups, generated using logistic regression (j), elastic net regression (k), and a multilayer perceptron (MLP) (l), all evaluated on test subjects.

The low-dimensional embeddings learned from SLOPE further went through UMAP to construct a two-dimensional progression trajectory, with all visits of training subjects ordered along a principal curve (Fig. 3a). The relative position of each visit on the principal curve was quantified as pseudotime, reflecting the global amyloid progression stage. Each visit was then assigned to a relative position along this curve, normalized to the range [0, 1], representing its pseudotime value (Fig. 3b).

We compared the distribution of pseudotime across diagnostic groups (Fig. 3c). As expected, CN participants exhibited significantly lower pseudotime values compared to both EMCI and LMCI groups ($p=1.3\times 10^{-17}$), while AD patients showed the highest pseudotime values overall ($p=3\times 10^{-24}$). However, there was no noticeable difference between the EMCI and LMCI groups in terms of pseudotime distribution. Importantly, when the visits of held-out test subjects were projected onto the same progression trajectory, we observed a consistent pattern of group separation (Fig. 3e–g), supporting the robustness and generalizability of the learned trajectory. Similar as SLOPE, we estimated pseudotime using an autoencoder without incorporating temporal information across follow-up visits. We observed a highly consistent pattern between the training and held-out test datasets, with an incremental pseudotime distribution from CN to MCI and then AD groups.

In contrast, the three supervised models—logistic regression, elastic net, and multilayer perceptron (MLP)—were trained as multi-class classifiers. To evaluate their ability to capture the progressive nature from CN to AD, we examined the distribution of their output logits (normalized to the [0,1] range) across diagnostic groups. Unlike SLOPE and the autoencoder, the normalized logits generated from the logistic regression and elastic net models showed statistically significant differences between diagnostic groups, but lacked a clear increasing trend from CN to MCI and AD. This suggests that while these models may distinguish between groups, they do not capture the progressive nature of amyloid accumulation. Among the supervised models, only the MLP produced logits that followed a broadly increasing pattern from CN to MCI to AD, partially reflecting the amyloid accumulation along disease progression.

### 3.2 Temporal consistency of follow-up visits in progression trajectory

We further evaluated the progression trajectory by assessing how well it preserved the temporal order of longitudinal follow-up visits. For participants with multiple visits, we expect the pseudotime to either remain stable or increase over time, reflecting the irreversible nature of amyloid accumulation. To quantify this, we computed two metrics: the violation ratio and the violation gap (as detailed in Section 2.4). Both metrics were calculated across a range of tolerance thresholds, which allow for the exclusion of minor fluctuations in pseudotime.

**Figure 4:**
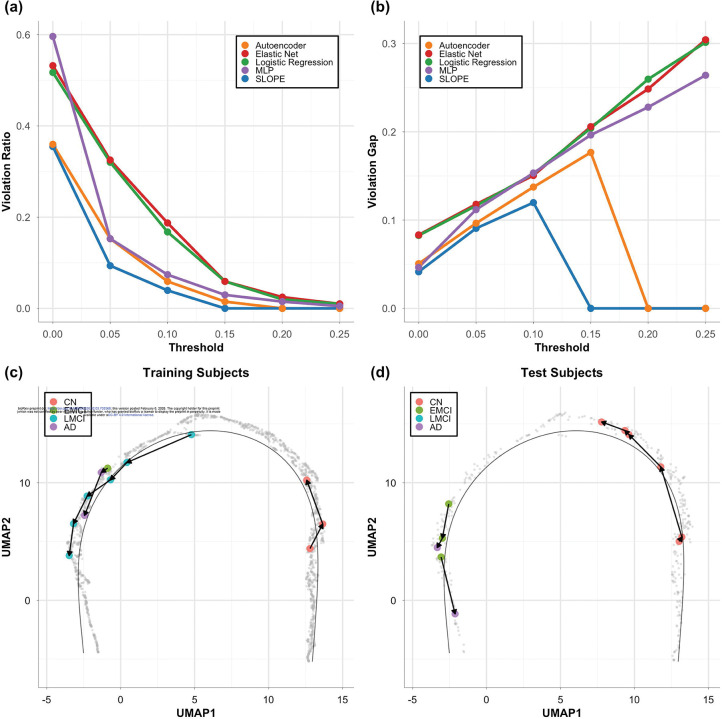
Temporal consistency across follow-up visits evaluated through violation ratio (a) and violation gaps (b). In (c) and (d) are a few example training and test subjects highlighted on the progression trajectory derived from SLOPE. Each dot is a visit, and each subject is represented as a sequence of connected follow-up visits.

Fig. 4a shows the violation ratio, which reflects the percentage of consecutive visits with pseudotime reversal exceeding a specified tolerance threshold, where the later visit has a pseudotime value lower than the earlier one. Fig. 4b presents the corresponding violation gap, which quantifies the average magnitude of those reversals. Across all tested thresholds, SLOPE consistently demonstrated the lowest violation ratio and smallest violation gap compared to both unsupervised and supervised baseline methods. At a tolerance threshold of 0.1, only approximately 4% of consecutive visits showed reversal in SLOPE-derived pseudotime, with an average violation gap of around 0.12. This is substantially lower than those observed in other methods, which often showed both higher frequencies of reversals and larger reversal gaps. Notably, SLOPE-derived pseudotime showed no reversals greater than 0.15, further highlighting its strength in preserving temporal consistency of follow-up visits.

Taking together, these results demonstrate the robustness of SLOPE in modeling disease progression in a temporally consistent manner, making it a strong candidate for tracking disease progression over time. In Fig. 4c–d we highlighted the follow-up visits from several example training and test subjects. This 2D progression trajectory enables direct visualization of individual-level progression dynamics, providing a practical tool for visually tracking the disease development of patients and monitoring the treatment outcomes in clinical settings.

### 3.3 Comparison with global SUVR in capturing early amyloid progression

**Figure 5:**
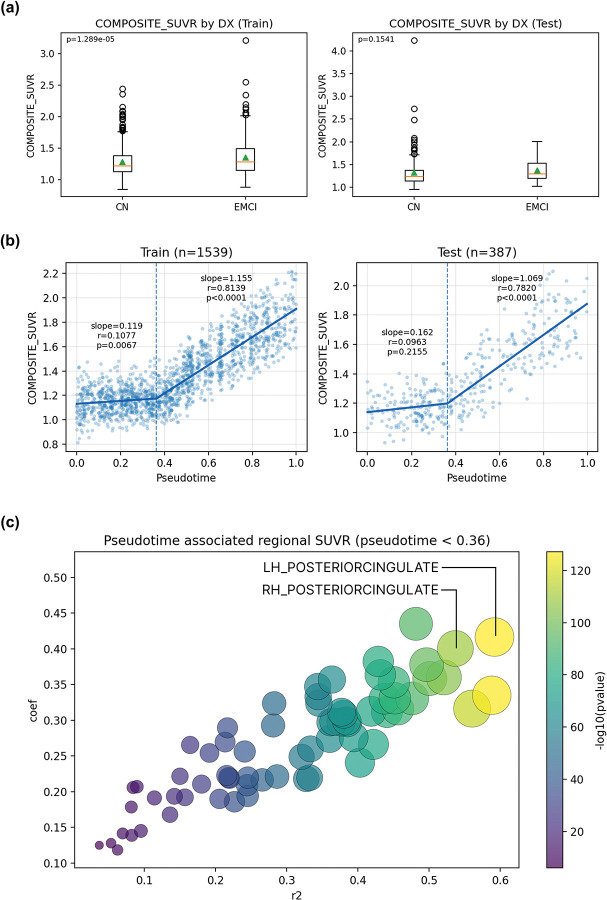
Pseudotime associated regional SUVR (pseudotime < 0.36). (a) Boxplots comparing composite SUVR between CN and EMCI groups for both train and test datasets. (b) Two-segments linear regression between SLOPE pseudotime and composite SUVR. (c) 68 MRI features correlations in early pseudotime.

In the training set, composite amyloid SUVR differed significantly between CN and EMCI groups ($p=1.29\times 10^{-5}$) (Fig. 5a), though this separation was weaker than that observed with SLOPE pseudotime ($p=1.3\times 10^{-17}$) (Fig. 3c). In the held-out test set, composite amyloid SUVR did not differentiate CN and EMCI groups (Fig. 5a), whereas SLOPE pseudotime continued to show a significant separation ($p=5.2\times 10^{-7}$) (Fig. 3f), indicating improved robustness for early-stage differentiation. Segmented regression revealed a nonlinear relationship between composite amyloid SUVR and SLOPE pseudotime (Fig. 5b). In both training and test datasets, composite SUVR remained relatively stable until pseudotime reached approximately 0.36, after which it became strongly correlated with pseudotime (r≈0.8). Regional analyses showed that early pseudotime variation (before 0.36) was driven by amyloid accumulation in specific cortical regions, most prominently the posterior cingulate cortex (Fig. 5c). [12, 13]

### 3.4 Brain changes along the progression trajectory

**Figure 6:**
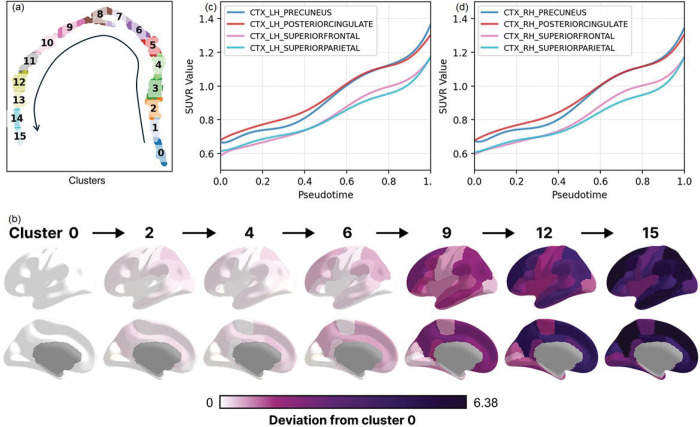
(a) Sixteen clusters were identified along the two-dimensional SLOPE-derived progression trajectory. (b) For each of the 68 bilateral cortical regions, we quantified the deviation of subsequent clusters relative to the earliest cluster (Cluster 0). (c, d) Early amyloid elevation was observed in the precuneus, posterior cingulate, superior parietal, and superior frontal cortices.

We performed a clustering analysis across all visits along the trajectory. This clustering analysis resulted in 16 clusters organized along the progression trajectory (Fig. 6a). The number of clusters was chosen for visualization purposes to provide sufficient granularity. Using Cluster 0 as the reference (representing the earliest stage), we computed the median regional SUVR values within each subsequent cluster and quantified their deviation from Cluster 0 using the following equation::

#(3) $$d\left(C_{i},C_{0}\right)=\frac{{\tilde{X}}_{i}-{\tilde{X}}_{0}}{\sigma_{0}}$$

Here, for each brain region, ${\tilde{X}}_{i}$ and ${\tilde{X}}_{0}$ represent the median SUVR values in Cluster i and Cluster 0, and $\sigma_{0}$ is the standard deviation of Cluster 0. This deviation captures how much the amyloid-β uptake levels in each brain region diverges from the baseline (Cluster 0) as the disease advances.

An initial noticeable elevation in amyloid-β uptake levels was observed in the posterior cingulate and precuneus (Fig. 6b).. Their overall amyloid uptake levels start high and start to increase rapidly from the very early stage. Both regions are core components of the default mode network (DMN), aligning with previous reports that DMN structures are particularly vulnerable to early amyloid deposition [13]. The amyloid burden within the superior frontal cortex also demonstrated a noticeable deviation from cluster 0 in the early stage. However, upon closer examination, we found that SUVR values in the superior frontal cortex remained substantially lower than those observed in the precuneus and posterior cingulate. This pattern may reflect contributions from its anteromedial and medial subregions, which are also part of the DMN and have been suggested to be among the first sites of amyloid accumulation in the preclinical stages of AD. Another core hub of default mode network, the inferior parietal cortex, also showed signs of early elevation in amyloid uptake level, as consistently implicated in previous studies [13]. Furthermore, the superior parietal lobe was found to undergo a relatively rapid increase in the early stage as well. But its overall amyloid uptake is substantially lower compared to early hubs such as the precuneus, posterior cingulate, and inferior parietal cortex (Fig. 6c–d). This suggests that while the superior parietal lobe is not among the first regions to accumulate amyloid, it can show fast early growth once deposition is initiated, reflecting its vulnerability as the pathology spreads outward from the core default mode network hubs. In contrast to regions within the default mode network, the precentral and postcentral cortices, together with the lateral occipital cortex, showed little deviation until approximately Cluster 9, suggesting that these areas did not experience substantial amyloid-β elevation until later stages of disease progression. This observation aligns with prior studies that have consistently identified these regions as relatively late in the amyloid accumulation sequence [14].

## 4. Discussion

In this work, we introduced SLOPE, a novel unsupervised dimension reduction technique to model the AD progression in a continuous time scale and applied it to capture the global amyloid accumulation process. It learns directly from longitudinal amyloid data and their temporal sequence information, without using any diagnostic labels. It orders all visits along a principal curve, yielding a compact and smooth two-dimensional progression trajectory that captures global changes in the brain amyloid profile. Importantly, this trajectory preserves not only the progressive pattern across diagnostic groups but also the temporal ordering of repeated follow-up visits, thereby reflecting both inter- and intra-individual patterns of disease progression.

Several key advantages distinguish SLOPE from existing methods. First, it is parametric and therefore enables the projection of unseen subjects onto the learned 2D trajectory. This makes SLOPE suitable not only for exploratory discovery but also for prospective applications such as monitoring progression in clinical trials. Second, in contrast to traditional unsupervised clustering methods that primarily separate groups, SLOPE additionally captures the temporal relationships between clusters, reflecting the irreversible nature of amyloid accumulation as the disease advances. Third, to our knowledge, SLOPE is the first unsupervised progression model to preserve the temporal sequence of follow-up visits, addressing a limitation of most dimensionality reduction or supervised learning techniques, which often treat repeated visits as independent samples. By preserving the temporal continuity across diagnosis groups and follow-up visits, SLOPE produces a progression trajectory that is both biologically meaningful and clinically trustworthy.

In addition, the differing behavior of composite amyloid SUVR and SLOPE pseudotime highlights an important distinction between global and regionally informed measures. Composite SUVR is dominated by regions with already high amyloid SUVR and therefore primarily reflects widespread amyloid burden at later disease stages. As a result, subtle regional changes during early progression may have little impact on the global average. In contrast, SLOPE integrates multiregional information and is trained to capture localized amyloid changes for differentiation of follow-up visits even when global composite SUVR remains relatively stable. This property further explains why SLOPE is able to capture early amyloid progression and longitudinal change that are not evident from global summary measures alone. Also, although pseudotime is scaled between 0 and 1 and does not correspond to calendar time, it provides a relative measure of amyloid pathology severity, with the learned ordering capturing biologically meaningful transitions along early amyloid progression.

Closer examination of the two-dimensional progression trajectory revealed a biologically consistent amyloid spreading pattern that aligns with established clinical observations. The earliest changes emerged in the posterior cingulate cortex and precuneus, both core hubs of the default mode network, which have been repeatedly reported as highly vulnerable to early amyloid accumulation [12, 15]. This concordance validates the inferred trajectory and reinforces the potential of SLOPE as a tool for in vivo disease staging and progression modeling. Moreover, the ability of SLOPE to recover known pathological patterns without supervision suggests its applicability in detecting novel progression signatures across other neurodegenerative diseases. By modeling AD on a continuous time scale, SLOPE enables the ordering of subjects and their follow-up visits along a disease trajectory, facilitating the identification of the earliest signs of amyloid deposition even in individuals who are globally amyloid-negative. Interpretation of the 2D progression trajectory further provides valuable insights into the sequence of regional amyloid initiation and accumulation, which could guide the design of amyloid composite scores sensitive for early detection, improve monitoring of progression, and inform the development of prevention strategies in AD.

Despite its strengths, this study has several limitations. First, the cohort was limited to an older population (primarily > 65 years), which limits the ability to characterize the very earliest stages of amyloid changes. This may partly explain why multiple brain regions appeared to show noticeable amyloid elevation simultaneously in the early clusters. Additional longitudinal data from younger cohorts will be important to improve our understanding of early-stage progression. Second, SLOPE was applied here solely to amyloid PET data. Incorporating other imaging modalities (e.g., tau PET and MRI) could provide a more comprehensive view of AD development and clarify the interplay between amyloid, tau, and neurodegeneration.

## Figures and Tables

**Table 1:** Demographic information of all participants.

DXGrp	CN	EMCI	LMCI	AD

Number	266	298	194	203
Gender	119/147	174/124	119/75	123/80
Baseline Age (mean±sd)	74.87±6.99	72.50±7.11	74.77±8.15	75.44±7.83
Years of Education (mean±sd)	16.53±2.50	16.10±2.65	16.09±2.82	15.78±2.65

**Table 2:** Comparison of classification performance (AD vs. CN) on held-out test subjects across different input feature sets and classification models.

	SLOPE embedding	AE embedding	Regional SUVR
	
	Logistic Regression		

Accuracy	**0.863**	0.853	0.794
F1-Score	**0.841**	0.828	0.753
AUC	**0.858**	0.847	0.785

	Elastic Net		

Accuracy	**0.853**	0.843	0.794
F1-Score	**0.828**	0.814	0.753
AUC	**0.847**	0.836	0.785

	MLP		

Accuracy	**0.853**	0.833	0.833
F1-Score	**0.828**	0.8	0.8
AUC	**0.847**	0.825	0.825

We evaluated three types of input features: original regional SUVR values, SLOPE-derived embeddings, and autoencoder (AE)-derived embeddings. The SLOPE and autoencoder embeddings are low-dimensional features from the output of their respective encoders.

## References

[1] Amieva H. , “Prodromal Alzheimer’s disease: successive emergence of the clinical symptoms,” Annals of Neurology: Official Journal of the American Neurological Association and the Child Neurology Society, vol. 64, no. 5, pp. 492–498, 2008 2008. 10.1002/ana.21509 19067364

[2] Brockmann R., Nixon J., Love B. L., and Yunusa I., “Impacts of FDA approval and Medicare restriction on antiamyloid therapies for Alzheimer’s disease: patient outcomes, healthcare costs, and drug development,” The Lancet Regional Health–Americas, vol. 20, 2023 2023. 10.1016/j.lana.2023.100467 PMC9996432 36908502

[3] Olatunde O. O. , “Multiclass classification of Alzheimer’s disease prodromal stages using sequential feature embeddings and regularized multikernel support vector machine,” NeuroImage, vol. 304, p. 120929, 2024 2024. 39571644 10.1016/j.neuroimage.2024.120929

[4] Li J. , “Roles of alternative splicing in modulating transcriptional regulation,” BMC Systems Biology, vol. 11, no. Suppl 5, p. 89, 2017 2017. 28984199 10.1186/s12918-017-0465-6 PMC5629561

[5] Street K. , “Slingshot: cell lineage and pseudotime inference for single-cell transcriptomics,” BMC Genomics, vol. 19, no. 1, p. 477, 2018 2018. 29914354 10.1186/s12864-018-4772-0 PMC6007078

[6] Qiu X. , “Reversed graph embedding resolves complex single-cell trajectories,” Nature methods, vol. 14, no. 10, pp. 979–982, 2017 2017. 28825705 10.1038/nmeth.4402 PMC5764547

[7] Moon K. R. , “Visualizing structure and transitions in high-dimensional biological data,” Nature biotechnology, vol. 37, no. 12, pp. 1482–1492, 2019 2019. 10.1038/s41587-019-0336-3 PMC7073148 31796933

[8] He B. , “Integrating amyloid imaging and genetics for early risk stratification of Alzheimer’s disease,” Alzheimer’s & Dementia, vol. 20, no. 11, pp. 7819–7830, 2024 2024. 10.1002/alz.14244 PMC11567859 39285750

[9] He B., Zhang S., Risacher S. L., Saykin A. J., and Yan J., “Multi-modal Imaging-based Pseudotime Analysis of Alzheimer progression,” 2024 2024: World Scientific, pp. 664–674. 10.1142/9789819807024_0047 PMC12044618 39670403

[10] Mukherjee S. , “Molecular estimation of neurodegeneration pseudotime in older brains,” Nature Communications, vol. 11, no. 1, p. 5781, 2020 2020. 10.1038/s41467-020-19622-y PMC7666177 33188183

[11] Frisoni G. B. , “Strategic roadmap for an early diagnosis of Alzheimer’s disease based on biomarkers,” The Lancet Neurology, vol. 16, no. 8, pp. 661–676, 2017 2017. 28721928 10.1016/S1474-4422(17)30159-X

[12] Ali D. G. , “Amyloid-PET levels in the precuneus and posterior cingulate cortices are associated with executive function scores in preclinical Alzheimer’s disease prior to overt global amyloid positivity,” Journal of Alzheimer’s Disease, vol. 88, no. 3, pp. 1127–1135, 2022 2022. 10.3233/JAD-220294 PMC10349398 35754276

[13] Palmqvist S. , “Earliest accumulation of β-amyloid occurs within the default-mode network and concurrently affects brain connectivity,” Nature Communications, vol. 8, no. 1, p. 1214, 2017 2017. 10.1038/s41467-017-01150-x PMC5663717 29089479

[14] Grothe M. J. , “In vivo staging of regional amyloid deposition,” Neurology, vol. 89, no. 20, pp. 2031–2038, 2017 2017. 29046362 10.1212/WNL.0000000000004643 PMC5711511

[15] Pfeil J. , “Unique regional patterns of amyloid burden predict progression to prodromal and clinical stages of Alzheimer’s disease,” Neurobiology of aging, vol. 106, pp. 119–129, 2021 2021. 34284259 10.1016/j.neurobiolaging.2021.06.014 PMC8461082

